# Effectiveness of genomic selection for improving provitamin A carotenoid content and associated traits in cassava

**DOI:** 10.1093/g3journal/jkab160

**Published:** 2021-05-08

**Authors:** Williams Esuma, Alfred Ozimati, Peter Kulakow, Michael A Gore, Marnin D Wolfe, Ephraim Nuwamanya, Chiedozie Egesi, Robert S Kawuki

**Affiliations:** 1 National Crops Resources Research Institute, Kampala, Uganda; 2 International Institute for Tropical Agriculture, Ibadan, Nigeria; 3 Plant Breeding and Genetics Section, School of Integrative Plant Science, Cornell University, Ithaca, NY 14853, USA

**Keywords:** breeding, carotenoids, genetic gain, genomic prediction, Manihot esculenta, vitamin A deficiency

## Abstract

Global efforts are underway to develop cassava with enhanced levels of provitamin A carotenoids to sustainably meet increasing demands for food and nutrition where the crop is a major staple. Herein, we tested the effectiveness of genomic selection (GS) for rapid improvement of cassava for total carotenoids content and associated traits. We evaluated 632 clones from Uganda’s provitamin A cassava breeding pipeline and 648 West African introductions. At harvest, each clone was assessed for level of total carotenoids, dry matter content, and resistance to cassava brown streak disease (CBSD). All clones were genotyped with diversity array technology and imputed to a set of 23,431 single nucleotide polymorphic markers. We assessed predictive ability of four genomic prediction methods in scenarios of cross-validation, across population prediction, and inclusion of quantitative trait loci markers. Cross-validations produced the highest mean prediction ability for total carotenoids content (0.52) and the lowest for CBSD resistance (0.20), with G-BLUP outperforming other models tested. Across population, predictions showed low ability of Ugandan population to predict the performance of West African clones, with the highest predictive ability recorded for total carotenoids content (0.34) and the lowest for CBSD resistance (0.12) using G-BLUP. By incorporating chromosome 1 markers associated with carotenoids content as independent kernel in the G-BLUP model of a cross-validation scenario, prediction ability slightly improved from 0.52 to 0.58. These results reinforce ongoing efforts aimed at integrating GS into cassava breeding and demonstrate the utility of this tool for rapid genetic improvement.

## Introduction

Cassava (*Manihot esculenta* Crantz), grown on approximately 18 million hectares in Africa, offers great potential to end extreme hunger, achieve food security and improve nutrition, if proven varieties are unconditionally accessed by producers and consumers (Kolawole *et al.* 2010). However, nutritionally cassava is deficient in essential micronutrients such as provitamin A carotenoids (Montagnac *et al.* 2009), which renders diets that heavily depend on the crop vulnerable to vitamin A deficiency (VAD). Indeed, VAD is a widespread nutritional challenge in sub-Saharan Africa, with women and children being the most affected (Gegios *et al.* 2010; Stephenson *et al.* 2010). For this reason, cassava varieties with elevated levels of provitamin A carotenoids are being developed and promoted (Pfeiffer and McClafferty 2007) as a cost-effective and sustainable approach to help communities burdened by VAD (Bouis *et al.* 2011; Talsma *et al.* 2013). The significant variation for total carotenoid content (TCC) (ranging from 1.02 to 10.4 µg g^−1^), of which >90% is constituted by ß-carotene, a provitamin A carotenoid, reported in large collection (>2400 clones) of cassava accessions (Chávez *et al.* 2005; Nassar *et al.* 2007) could facilitate the effective breeding of nutrition-sensitive varieties.

A major drawback in our endeavors to breed cassava varieties with elevated provitamin A carotenoids has been the undesirable tendency of low dry matter content (DMC) in most provitamin A cassava clones (Njoku *et al.* 2015; Esuma *et al*. 2016a). Another worrisome and unique challenge to cassava breeding in East Africa is the menace of cassava brown streak disease (CBSD) caused by two *Ipomoviruses*: *Ugandan cassava brown streak virus* and *Cassava brown streak virus* (Alicai *et al.* 2016; Kawuki *et al.* 2016). Collectively, these conundrums of low DMC and CBSD that directly affect root quality hamper speedy development and dissemination of provitamin A cassava clones in eastern Africa. It is for such situations that tools for implementing efficient crop improvement programs to address intricate breeding obstacles are being optimized (Ahmar *et al.* 2020).

For example, the rapid advances in next generation sequencing (NGS) technologies have enabled the use of genome-wide markers for implementing genomic selection (GS), a tool that can significantly enhance the efficiency of crop breeding (Bhat *et al.* 2016). Essentially, GS aims to increase the speed and accuracy of selection in breeding programs by predicting the genetic value of individuals or lines at an early selection stage, or for individuals that cannot be directly phenotyped (Crossa *et al.* 2017). One of the salient features of GS is the use of high-density markers for prediction, which relies on genotyping at a high marker density to ensure most causal loci are in linkage disequilibrium (LD) with at least one marker (Jannink *et al.* 2010). By using dense markers to quantify Mendelian sampling, GS avoids the need for extensive progeny phenotyping, which saves cost and time by reducing the length of a breeding cycle, while enhancing genetic gain per selection cycle (Desta and Ortiz 2014). Consequently, GS can facilitate rapid crop improvement for relevant agronomic and end-user traits, as witnessed in different crops (Xu *et al.* 2018, 2020).

A broad review of GS in plant breeding, detailing requirements for training population and features of prediction models, has been elegantly provided by Desta and Ortiz (2014). In fact, response of GS in crop improvement may be affected by factors such as model performance, sample size and relatedness, marker density, heritability and genetic architecture of traits, and the extent and distribution of LD between markers and quantitative trait loci (QTL) on the accuracy of genomic estimated breeding values (GEBVs) (Zhong *et al.* 2009). Thus, predictive accuracy varies among GS models depending on their assumptions and treatments of marker effects (Ornella *et al.* 2012).

Indeed, results from both simulation and empirical studies have illustrated the efficacy and limitations of GS for several crops, including maize (Bernardo and Yu 2007), rice (Xu *et al.* 2018), barley (Nielsen *et al.* 2016), and wheat (Kristensen *et al.* 2018). Wolfe *et al.* (2017) reported some promising results depicting prospects for GS in cassava, with higher prediction accuracies for DMC (0.36–0.48) and cassava mosaic disease (CMD) (0.26–0.40) than those for fresh root yield attributes (<0.1). Relatedly, Kayondo *et al.* (2018) evaluated the accuracy of seven genomic prediction models using empirical data from 1301 cassava clones and reported predictive ability of 0.31–0.42 for CBSD severity in roots, indicating GS as a useful tool for CBSD resistance breeding. Furthermore, Ozimati *et al.* (2018) reported increased genomic prediction accuracies for CBSD resistance arising from optimized training populations and the prospect of East African training sets to predict CBSD in West African cassava germplasm.

Motivated by these promising results from the pioneering efforts on the application and use of GS in cassava breeding, our study was aimed at exploring genomic prediction as a strategy for the rapid development of carotene-rich cassava product profiles suited for the growing conditions in East Africa. Broadly, this study was part of an ongoing effort to optimize and integrate GS into the provitamin A cassava breeding scheme in Uganda for rapid genetic improvement. The provitamin a cassava breeding pipeline currently utilizes locally adapted germplasm (Esuma *et al*. 2016a), with occasional introductions and introgression from the West African germplasm, where remarkable success has been achieved in cassava biofortification. Thus, we evaluated genomic prediction ability within the base population for provitamin A carotenoids and assessed the effectiveness of this population to predict performance of subsequent breeding cycles and that of West African germplasm. Specifically, we tested (i) the predictive ability for TCC, DMC, and CBSD across different statistical models and (ii) the utility of QTL markers in genomic prediction for TCC.

## Materials and methods

### Plant materials

Three sets of germplasm were used in this study. First, a panel of 280 clones segregating for root quality and agronomic attributes were selected from a cassava breeding population at National Crops Resources Research Institute (NaCRRI), Uganda. This panel constituted the training population, hereafter referred to as Cycle 0. Second, a subset of 50 Cycle 0 clones with the highest breeding values for carotenoid content was identified (through cross-validation described hereafter) as progenitors and planted in a crossing nursery at Namulonge to generate cycle one (Cycle 1) population, during the period 2016/2017. Following seed germination and preliminary screening at nursery and seedling stages, 352 Cycle 1 genotypes were cloned and evaluated in 2018. Third was a set of 648 clones derived from 80 progenitors introduced from West Africa (WA) through International Institute for Tropical Agriculture (IITA); this population segregated for carotenoids and DMC.

### Field trials and phenotypic data collection

The Cycle 0 population was evaluated at two locations, Namulonge (0.52166458°N, 32.608997564°E) and at Serere (1.4994°N, 33.5490°E) during the 2017/2018 cropping season in Uganda. Both locations are characterized by high prevalence of CBSD causing viruses and high whitefly vector populations (Alicai *et al*. 2019) known to transmit such viruses. Cycle 1 and WA populations were evaluated at one location (Namulonge), due to the limitation of planting material. Trials were laid out in augmented design with ∼25–30 plots per block and four checks. Each plot was represented by a single row of 10 plants spaced at 1 × 1 m. In all trials, CBSD susceptible cultivar TME 204 was planted as spreader rows to provide source of virus inoculum.

At harvest (12 months after planting), all plants in a plot were uprooted. Total carotenoid content was assessed by visually scoring the intensity of pigmentation of the root parenchyma on a qualitative scale of 1–8 (Sánchez *et al.* 2006). We used the visual color scale for carotenoids content as it was the available high throughput phenotyping method the time of data collection and previous reports have indicated a strong positive correlation between carotenoid content assessed visually and quantitatively (Esuma *et al*. 2016a). Furthermore, all roots harvested per plot were pooled and screened for CBSD necrosis severity using a scale of 1–5 described by Hillocks and Thresh (2000), where: 1 = no necrosis, 2 = ≤5% of the root is necrotic, 3 = 6–10% of the root is necrotic, 4 = 11–25% of the root is necrotic and mild root constriction and 5 = >25% of the root necrotic and severe root constriction. DMC was estimated from approximately 200 g of fresh root samples that were oven-dried into a constant weight at 105°C for 24 hours. Subsequently, DMC was computed using the formula:

$$\text{DMC}\left(\%\right)=\frac{\text{DSW}}{\text{FSW}}\times 100$$

where DSW = dry sample weight and FSW = fresh sample weight.

### Genotyping of samples using DArTseq technology

Leaf tissues were collected from all test clones evaluated and sent to Intertek and Diversity Array Technology Pty Ltd. (http://www.diversityarrays.com/) for DNA extraction and genotyping, respectively. Briefly, DArTseq technology relies on a complexity reduction method to enrich genomic representations with single copy sequences and subsequently perform next-generation sequencing using HiSeq 2500 (Illumina, USA). Further detail of the DArTseq genotyping process has been described by Kilian *et al.* (2012).

In our case, sequences of the genomic representations were aligned to cassava reference genome v6.1. Eventually, 13,675 high-quality single nucleotide polymorphism (SNP) markers were selected using the following quality control parameters (Kilian *et al.* 2012): (i) the reproducibility of 100%, (ii) the overall call rate over 95%, and (iii) the polymorphic information content between 0.3 and 0.5. Genotypes were coded as 0 (homozygous for reference allele), 1 (heterozygous), and 2 (homozygous SNP). Genotype data were imputed to a panel of 23,431 SNPs using the Beagle 5.0 algorithm (Browning *et al.* 2018) and a reference panel of 20,733 mostly East African cassava haplotypes, derived from a combination of genotyping-by-sequencing and DArTseq-LD. The data are available here: ftp://ftp.cassavabase.org/marnin_datasets/nextgenImputattion2019/.

### Statistical analyses

#### Estimates of variance components and heritability:

Phenotypic dataset for each trial was considered independent and analyzed separately using a two-step genomic prediction approach. In the first step, we fitted linear mixed models accounting for each trial’s design and extracted the best linear unbiased predictions (BLUPs) of the clone effects for TCC, DMC, and CBSD root necrosis using the *lme4* package for R statistical software (Bates *et al.* 2015). For Cycle 0 that was evaluated in two locations, we fitted a model $y=X\beta +Z_{g^{c}}+Z_{{\mathrm{block}(l)}^{b}}+Z_{{g.l}^{l}}+\varepsilon$, where vector β was the fixed effect for grand mean and location, with the corresponding incidence matrix *X*; vector *c* and corresponding incidence matrix *Z_g_* was the random effect for clones (*g*) such that$c∼N\left(0,I\sigma_{c}^{2}\right)$; vector *b* with its corresponding incidence matrix *Z_block(l)_* represented random effect for blocks nested in locations (*l*) such that $b∼N\left(0,I\sigma_{b}^{2}\right);$vector *l* and incidence matrix *Z_g.l_* represented random effect of genotype-environment interaction; and εwas the residual such that$e∼N\left(0,I\sigma_{e}^{2}\right)$. For Cycle 1 clones and the West African introductions, each evaluated in one location, we fitted a linear mixed model $y=X\beta +Z_{g^{c}}+Z_{{\mathrm{block}}^{b}}+\varepsilon$ wherey was the vector of raw phenotypes, β was a fixed effect of grand mean, and $Z_{{\mathrm{block}}^{b}}$ represented the random effect for blocks.

Variance components were extracted from the models for estimation plot-based heritability (*H*) as:

$$H=\frac{{\sigma^{2}}_{c}}{{{(\sigma }^{2}}_{c}{{+\sigma }^{2}}_{\text{cl}}+{\sigma^{2}}_{e})}$$

where ${\sigma^{2}}_{c}$was the clone variance, ${\sigma^{2}}_{\text{cl}}$ was the variance attributed to genotype by location interaction (excluded for trials conducted in one location) and ${\sigma^{2}}_{e}$was the model residual variance. Furthermore, we used the *mixed.solve* function in R to fit a single-stage genomic best linear unbiased prediction (G-BLUP) model, with the grand mean and location as fixed effects and clone effects treated randomly. Eventually, SNP-based heritability ($h^{2})$was computed as:

$$h^{2}=\frac{{\sigma^{2}}_{a}}{{{(\sigma }^{2}}_{a}+{\sigma^{2}}_{e})}$$

where ${\sigma^{2}}_{a}$clone was the additive genetic variance and ${\sigma^{2}}_{e}$was the residual variance.

The total genetic value of each individual was estimated as BLUP extracted from the mixed linear models following the procedure described by Garrick *et al.* (2009). To avoid applying shrinkage to the same data twice (at the first step and subsequent genomic prediction step), the BLUPs were de-regressed as:

$$\text{de}-\text{regressed}\text{BLUP}=\frac{\text{BLUP}}{1-\frac{\text{PEV}}{{\sigma^{2}}_{c}}}$$

where PEV represented the prediction error variance for the BLUPs and ${\sigma^{2}}_{c}$ was the clone variance. The de-regressed BLUPs were used in subsequent genomic prediction analyses.

#### Population structure:

To assess population structure, we used 23,431 polymorphic DArTSeq markers filtered to keep only SNPs with minor allele frequency (MAF) ≥ 0.01. Using the *A.mat* function built in the rrBLUP R package (Endelman 2011), we constructed a realized genomic relationship matrix (*K*) from SNP data. Finally, principal component analysis (PCA) was done on the genomic relationship matrix, using the *prcomp* function in R. The first two principal components (PC1 and PC2) were used to visualize population structure.

#### Estimating the ability of genomic prediction models through cross-validation:

We used the BLUPs in a fivefold cross-validation scheme, with 10 replications, to evaluate prediction ability for TCC, DMC, and CBSDrs across four parametric GS models: (i) genomic BLUP (G-BLUP), (ii) BayesA, (iii) BayesB, and (iv) Bayesian Lasso. The main features of these genome-wide prediction models have been reviewed by Desta and Ortiz (2014). To perform cross-validation with G-BLUP, the *A.mat* function in the R package rrBLUP was initially used to construct a genomic realized relationship matrix from SNP marker dosages and GEBVs obtained after fitting a linear mixed model using the *mixed.solve* function in the same package. Cross-validations for Bayesian models were computed with the BGLR package (Perez and de los Campos 2014), with model parameters *nIter* and *burnIn* fixed at 10,000 and 1000, respectively.

To achieve cross-validation for each fold in a replication, the total number of genotypes in a population was divided into five equal proportions such that four groups at a time formed the training set to build the prediction model, while the fifth group was the test set. For example, when cross-validation was performed for Cycle 0 (*n* = 280), 224 genotypes were used as training set while the remaining 56 individuals were used as validation set. This process was repeated for each of the five folds across 10 replications. Prediction abilities were computed as Pearson’s correlation coefficients (*r*) between GEBVs predicted for the test set and the corresponding BLUPs obtained from the first step of the analysis.

#### GWAS-based cross-validation:

The purpose of GWAS analysis was to identify chromosome markers associated with each phenotype and use such information to design kernel-based cross-validation schemes. Thus, we surveyed for QTL for all the three traits using genome-wide association study (GWAS), using 632 individuals (constituted by Cycle 0 and Cycle 1) and 23,431 polymorphic markers filtered at MAF >0.05. To avoid any potential upward bias of prediction abilities, we performed GWAS in the training set of each fold across 10 replications of the cross-validation scheme, such that kernel-based predictions were done in the test set of the respective fold. For example, for each fold, ∼506 genotypes (training set) representing 80% of the 632 individuals were used for GWAS and the remaining ∼126 (test set) used for kernel-based predictions.

Specifically, GWAS was implemented using a linear mixed model in the R package *rrBLUP* following the modified methodology described by Isidro-Sánchez *et al.* (2017). The linear mixed model fitted included a kinship matrix and the first three principal components to account for population structure. We generated quantile-quantile and Manhattan plots generated with the R package *qqman* (Turner 2014) and used them to evaluate the association mapping model and chromosome-wise association signals such that SNPs with *P*-values less than the 5% Bonferroni threshold were considered to be significantly associated with phenotypes.

Based on DArTseq markers used in this study, GWAS results for DMC and CBSD did not show significant association signals. However, we identified one QTL for TCC on chromosome 1, which was consistently the same for each of the five folds across the 10 replications, with ∼49 markers meeting the Bonferroni threshold of association significance (Supplementary Figure S1). Subsequently, we designed four GWAS-guided cross-validation schemes for TCC: (a) single-kernel with significant QTL markers on chromosome 1, (b) single-kernel with all chromosome 1 markers, (c) single-kernel excluding all markers on chromosome 1, and (d) multi-kernel model that included the significant QTL markers and the rest of the markers fitted as independent kernels in G-BLUP model (Morota and Gianola 2014). Finally, we performed GWAS with all the 632 individuals and used the output to predicted TCC in the West African population along the four prediction scenarios.

### Data availability

All the raw phenotypic and genotypic data are available at the link provided for references. Supplementary material is available at figshare: https://doi.org/10.6084/m9.figshare.12752405.

## Results

### Heritability estimates and population structure

Total carotenoids content had the highest broad-sense, varying between 0.68 and 0.73, while CBSDrs had the lowest estimates (0.36–0.50) (Table 1). We did not record DMC in Cycle 1 clones due to insufficient quantity of roots arising from small plot sizes. Similar trends were observed for narrow-sense (SNP-based) heritability estimates, with the highest values (0.61–0.71) recorded for TCC and lowest (0.29–0.40) for CBSDrs (Table 1).

**Table 1:** Heritability estimates for traits measured at the clonal evaluation stage

Population	Number*^a^*	*H^b^*			*h^2c^*		
		TCC	DMC	CBSDrs	TCC	DMC	CBSDrs
C0_pVAC	280	0.68	0.58	0.46	0.64	0.55	0.40
C1_pVAC	352	0.73	—	0.50	0.61	—	0.29
WA	648	0.73	0.56	0.36	0.69	0.54	0.34

a Number of clones evaluated.

b Broad sense heritability.

c Narrow sense heritability; TCC, total carotenoid content; DMC, dry matter content; CBSDrs, cassava brown streak disease severity in roots. We did not analyze DMC in C1 due to insufficient quantity of roots arising from small plot sizes and high CBSD root necrosis.

**Figure 1:**
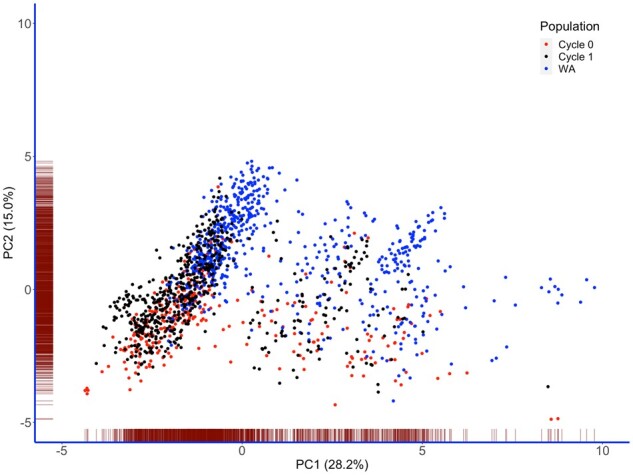
Plot of PC1 against PC2 for eigenvalue decomposition of DArTSeq marker genotypes for three sets of clones evaluated in this study.

PCA was used to describe population structure in the genetic materials analyzed. Grouping clones by the first two PCs (PC1 and PC2) showed no clear-cut differentiation among the three populations (Figure 1), with the PCs explaining 28.2 and 15% of the total genetic variation, respectively. Nonetheless, some WA clones tended to drift from other populations along PC2 (Figure 1).

### Cross-validation prediction accuracies for TCC, DMC, and CBSDrs

**Figure 2:**
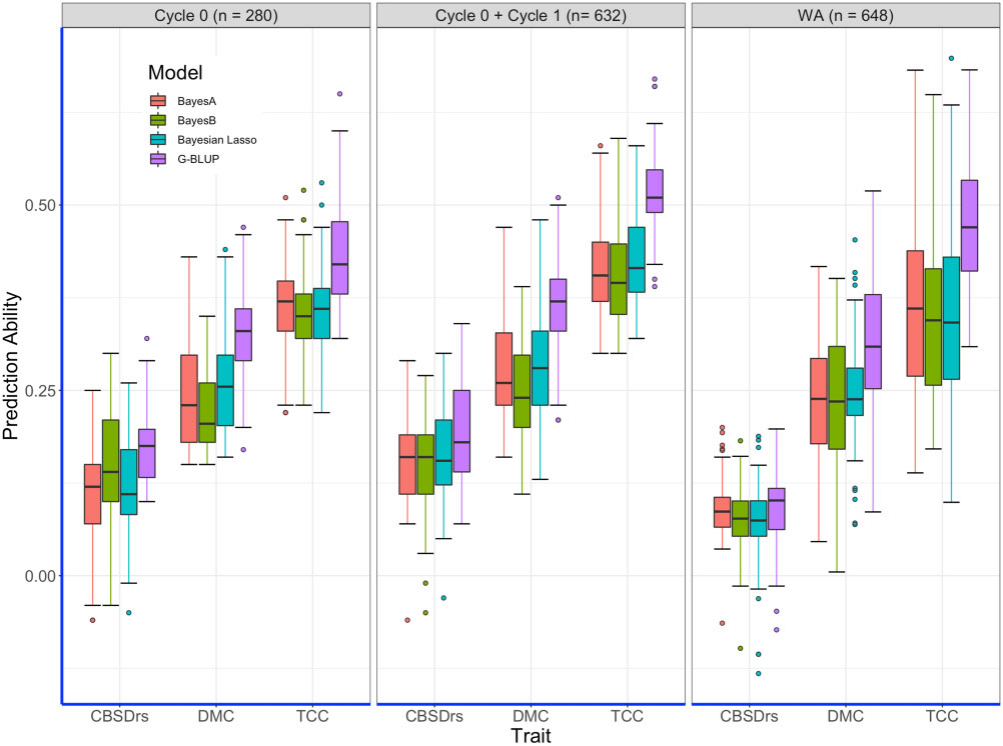
Prediction accuracies of five folds across 10 replications for all models evaluated in cross-validation schemes.

We performed cross-validation under three scenarios: within (a) Cycle 0 (*n* = 280 genotypes), (b) Cycle 0 combined with Cycle 1 (*n* = 632 genotypes), and (c) the West African population (*n* = 648 genotypes). Distribution of predictive abilities across the five folds and ten replications is presented in Figure 2, with an overall trend showing the highest predictive ability for TCC and the lowest for CBSDrs. For the cross-validation performed within Cycle 0, we recorded the highest mean prediction ability for TCC across all models, varying from 0.35 for BayesB and Bayesian Lasso to 0.44 for G-BLUP (Supplementary Table S1). Across all traits, G-BLUP had the highest mean predictive ability compared to the rest of the models tested across all three traits.

In the second cross-validation scenario that combined Cycle 0 combined with Cycle 1 clones, mean increase of 12.1% in predictive ability was recorded for TCC, with the highest increase (20%) recorded for Bayesian Lasso (Supplementary Table S1). In this scenario, the highest mean prediction ability of 0.52 was recorded for TCC with G-BLUP while the lowest value of 0.39 was noted for the trait with BayesB. Similarly, increase in training population size resulted into an increase of prediction ability for CBSDrs, except for BayesB that had no appreciable (0%) change.

Cross validation within the West African population showed similar trend, with the highest predictive ability 0.47 recorded for TCC with G-BLUP. For this population, predictive abilities for CBSDrs were remarkably low compared to values recorded in Cycle 0 and with Cycle 1.

### Across population prediction accuracies for TCC, DMC, and CBSDrs

We predicted each of the three traits across the four models under the following scenarios: Cycle 0 to predict performance of Cycle 1 and West African (WA) clones and a combination of Cycle 0 and with Cycle 1 to predict WA clones. First, we noted generally higher cross-population predictive ability for TCC than for other traits across all models, with the highest values recorded with G-BLUP. For example, the highest predictive ability of 0.43 was recorded for TCC when using the Cycle 0 to predict the performance of Cycle 1 (Table 2). However, when the Cycle 0 and Cycle 1 were combined to predict TCC in WA population, the predictive ability was relatively low (0.27) compared to the value of 0.34 obtained when the prediction was performed with Cycle 0 alone. A similar trend of low predictive ability in the WA population was noted for TCC across the other three models, with the lowest value (0.16) recorded with Bayesian Lasso in the scenario.

**Table 2:** Mean prediction abilities for across population predictions for TCC, DMC, and CBSDrs

hba	Testing set	Trait	G-BLUP	BayesA	BayesB	BL
Cycle 0	Cycle 1	TCC	0.431	0.392	0.411	0.401
Cycle 0	Cycle 1	CBSDrs	0.117	0.052	0.031	0.052
Cycle 0	WA	TCC	0.401	0.216	0.223	0.247
Cycle 0	WA	DMC	0.342	0.282	0.274	0.251
Cycle 0	WA	CBSDrs	0.045	0.019	0.014	0.009
Cycle 0 + Cycle 1	WA	TCC	0.274	0.162	0.161	0.159
Cycle 0 + Cycle 1	WA	CBSDrs	0.001	−0.123	−0.107	−0.103

DMC = of provitamin A cassava breeding population; dry matter content; CBSDrs, cassava brown streak disease severity in roots; BL, Bayesian Lasso.

Predictive abilities for CBSDrs were particularly low in all scenarios. For instance, the highest predictive ability (0.12) was only recorded when Cycle 0 was used to predict CBSDrs in Cycle 1 with G-BLUP; other models had negligible predictions for CBSDrs (Table 2). In fact, when Cycle 0 was used to predict CBSDrs in WA, all results were close to zero, with the highest predictive ability recorded was 0.05 for G-BLUP (Table 2). Lastly, we only tested the scenario of Cycle 0 predicting DMC in WA, as this trait was not assessed in Cycle 1. In this case, the highest predictive ability was 0.34 with G-BLUP and the lowest was 0.25 from Bayesian Lasso (Table 2).

### GWAS-guided genomic prediction

**Figure 3:**
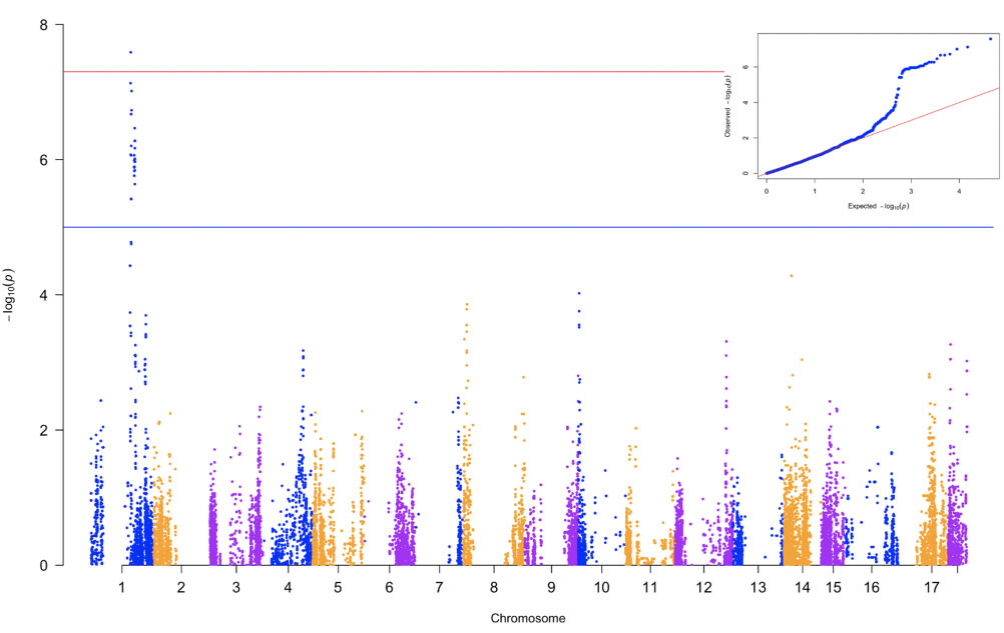
Manhattan plot for GWAS for total carotenoids content performed with 632 clones (constituted by Cycle 0 and Cycle 1). The horizontal blue line is the genome-wide significance (Bonferroni threshold); inset is the Q-Q plot for total carotenoid content.

**Figure 4:**
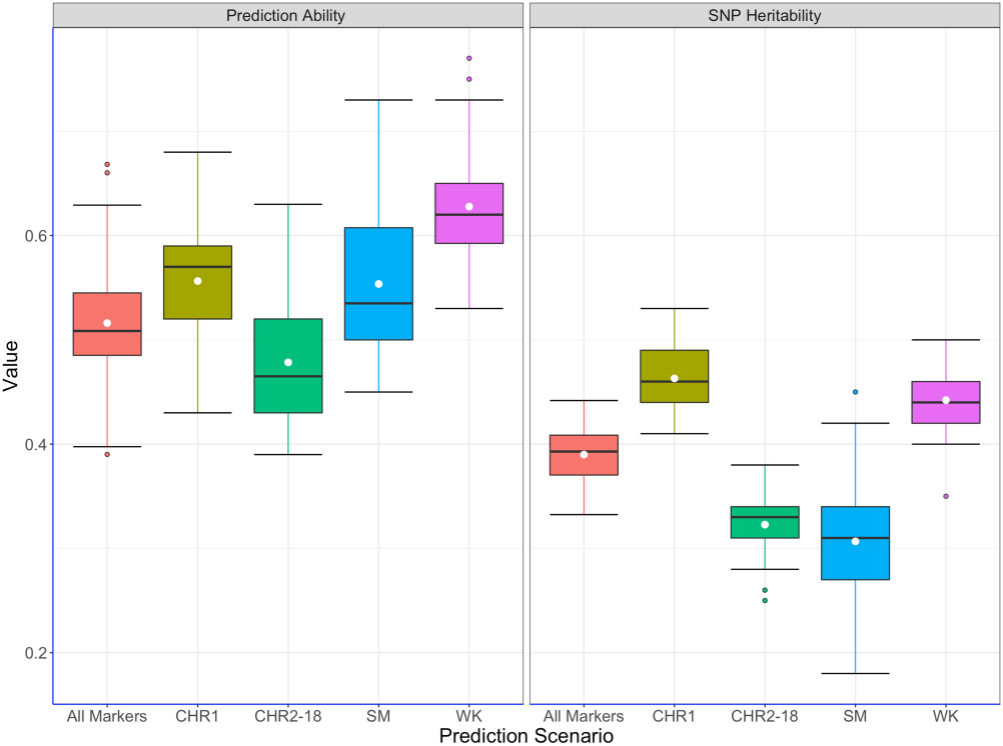
Prediction abilities for GWAS-guided scenarios for total carotenoids content for within-sub population cross-validation scheme. SM = scenario of predicting with significant QTL markers on chromosome 1 as single-kernel; CHR1 = scenario of predicting with all chromosome 1 markers as single-kernel; CHR2-18 = scenario of predicting with markers on chromosomes 2–18 only; WK = scenario of predicting with significant QTL markers on chromosome 1 and the rest of the markers as independent kernels.

All marker-trait associations for DMC and CBSDrs were nonsignificant (data not shown). GWAS for each training set per fold consistently revealed one QTL for TCC on chromosome 1, with ∼49 markers meeting the Bonferroni threshold for genome-wide association signal (Figure 3 and Supplementary Figure S1). Subsequently, GWAS-based prediction for TCC was performed in the respective test sets for each fold using cross-validation scheme with G-BLUP, which presented the highest prediction abilities compared to other models tested. The mean predictive abilities for the four scenarios of GWAS-guided cross-validation were 0.56 for single-kernel containing all chromosome 1 markers, 0.54 for single-kernel with significant chromosome 1 markers, 0.48 for single-kernel excluding all markers on chromosome 1, and 0.63 for the multi-kernel model that included the significant QTL markers and the rest of the markers fitted as independent kernels (Figure 4 and Supplementary Table S2). These scenarios led to better predictions than the case with naive model *i.e.*, without segmentation of chromosome markers, with the exception where single kernel excluding all chromosome 1 markers was used. Narrow-sense heritability estimates were proportionately higher for scenarios of predicting with markers on chromosome 1 and the multi-kernel approach than for the case of excluding chromosome 1 (Figure 4). When the GWAS-guided approach was used to predict TCC in WA, we noted similar pattern of the highest predictive ability (0.64) with multi-kernel scenario and the least (0.25) for single-kernel excluding chromosome 1 markers (Supplementary Table S2).

## Discussions

The rapid increase in the global human population, predicted to reach 10 billion by 2050 (Bongaarts 2009; Henderson and Loreau 2019), highlights the urgent need for deployment of high yielding, resilient and nutritious crop varieties to vulnerable societies (Ray *et al.* 2013; Hickey *et al.* 2019). The nutritional impacts of biofortification evidenced through staples like orange-fleshed sweet potato (Jenkins *et al.* 2015) provide motivation for development of cassava enriched with provitamin A carotenoids as nutritious staple in sub-Saharan Africa. Collectively, TCC, DMC, and CBSD resistance are must-have traits for product profiles targeting deployment of provitamin A cassava varieties in eastern Africa.

Breeding efforts, including our current results, have indicated moderate to high broad-sense heritability (0.56–0.73) for TCC and DMC, implying the possibility of making meaningful genetic gains for both traits through phenotypic recurrent selection (Ceballos *et al.* 2013; Esuma *et al*. 2016b). Indeed, the high heritability for TCC has already facilitated the identification of cassava cultivars with high levels of ß-carotene, which constitutes the bulk of carotenoids in cassava, with some varieties released for on-farm production in West Africa (Ayinde *et al.* 2017; Eyinla *et al.* 2019). However, the high costs and drudgery associated with phenotyping cassava roots for TCC and DMC (Ceballos *et al.* 2013), the apparent negative correlation between TCC and DMC (Njoku *et al.* 2015; Esuma *et al*. 2016b), and the unique challenge of CBSD in eastern and southern Africa (Alicai *et al.* 2016, 2019) were a major motivation for testing the prospects for GS as a tool for improving these traits in our provitamin A cassava breeding pipeline.

We found relatively low broad-sense heritability (0.36–0.50) for severity of CBSD root necrosis, which relates to the trait’s quantitative nature (Kulembeka *et al.* 2012; Masumba *et al.* 2017) strongly influenced by environmental factors, which complicates selection (Pariyo *et al.* 2015). However, heritability estimates above 0.5 have been reported by Kayondo *et al.* (2018) and Ozimati *et al.* (2019) for CBSD necrosis in populations evaluated for GWAS (1300 clones) and GS (922 clones), respectively. As expected, we noted lower estimates of SNP-based heritability for the three traits compared to the respective broad-sense heritability (Table 1). This could be attributable to under-tagging of causal loci by markers in our analysis (de los Campos and Toro 2017). Ozimati *et al.* (2019) observed similar trends for SNP-based heritability for several agronomic traits in a panel of >1000 clones evaluated for GS.

Based on the cross-validation results, predictive abilities were largely consistent across traits and prediction models. First, we noted a downward pattern for predictive ability TCC > DMC > CBSDrs across all models, and for both scenarios of small and increased size of training set. TCC is largely a qualitative trait (Welsch *et al.* 2010) for which genomic prediction is expected to be an effective tool (Zhang *et al.* 2019). Mean predictive abilities for DMC were generally low, ranging between 0.21 and 0.33, similar to the range of 0.29–0.34 reported by Wolfe *et al.* (2017) and Ozimati *et al.* (2018). Interestingly, in the same report, Wolfe *et al.* (2017) showed high predictive ability for DMC (0.63–0.67) in a cassava breeding population of IITA. The discrepancies in predictive abilities for DMC across breeding populations may relate to variation in heritability arising from differences in phenotyping methods of using specific gravity and oven-drying method and/or genetic differences of clones evaluated. Subsequently, efforts are being made to optimize high throughput methods like near infra-red spectroscopy for efficient phenotyping of root quality traits (Belalcazar *et al.* 2016; Ikeogu *et al.* 2017), which could improve reliability of GS for improving such traits.

Predictive abilities for CBSD resistance were generally low for all models tested in our analyses (mean of 0.20 for G-BLUP). Kayondo *et al.* (2018) highlighted GS as a promising tool to increase genetic gains for CBSD resistance in cassava, especially for nonparametric models like Random Forest and reproducing kernel Hilbert spaces regression, which capture both additive and nonadditive effects. It should be noted that Kayondo *et al.* (2018) used 1301 clones from NaCRRI’s white-fleshed cassava breeding population phenotyped in at least three environments, which probably presented robust data for genomic prediction. Nonetheless, introgressing CBSD resistance into the provitamin A cassava breeding population could increase the genetic merit of these clones for CBSD resistance and their use in developing varieties relevant for production in farmer’s fields.

When we explored prospects of cross-population predictions, we noted higher predictive ability across Uganda’s populations than in situations of predicting performance of WA clones. In evaluating the potential of East African training populations for genomic prediction of CBSD resistance in West African cassava germplasm, Ozimati *et al.* (2019) reported low predictive ability for the trait. Previous studies have indicated higher reliability of genomic predictions in populations that share ancestry with training sets than in situations of diverged genetic backgrounds where marker effects are likely to be different (De Roos *et al.* 2009; Lee *et al.* 2017). Indeed, a diagnosis of the second axis of PCA plot presented in Figure 1 shows an apparent drift of WA clones from the Ugandan clones, which is a manifestation of genetic difference. Nonetheless, this study revealed some possibility of predicting TCC and DMC in WA population using our cassava breeding population, with mean of 0.4 and 0.34, respectively, with G-BLUP. In this case, it would be possible to identify WA clones with superior GEBVs for TCC and DMC for further recombination with provitamin A cassava clones, which is a clever strategy to update a training set with useful alleles from external gene pools without disrupting genetic gain (Berro *et al.* 2019). However, the low-predictive ability for CBSDrs in the WA population could indicate low genetic merit of using WA cassava germplasm for CBSD resistance breeding, which reinforces the need for CBSD pre-emptive breeding as a precautionary measure for the accidental introduction and spread of CBSD in WA.

Motivated by success attained by selection programs that exploit GWAS-aided prediction (Li *et al.* 2019), we assessed the suitability of this method for our GS scheme for provitamin A cassava. In this case, we noted increased predictive ability for TCC by incorporating its QTL as an independent kernel in the genomic prediction model. The QTL for TCC identified in this study is within the genomic vicinity of *Manes.01G124200.1*, a gene which encodes enzyme phytoene synthase known to catalyze accumulation of ß-carotene in plant tissues (Welsch *et al.* 2010; Kandianis *et al.* 2013). In our study, the QTL represented biological information in the form of large-effect SNPs, which can enhance genomic prediction when fitted as an independent kernel. In this case, partitioning genomic markers into two relationship matrices, one kernel comprising the QTL markers associated with TCC on chromosomes 1 and the rest of the markers as the second kernel, led to better estimation of GEBVs (Morota and Gianola 2014). Li *et al.* (2019) reported increase in prediction accuracies of GS models resulting from increase in the number of most significant SNPs fitted as fixed effects in a maize breeding population. The apparent increase in predictive ability in our scenario of fitting effects of significant QTL markers on chromosome 1 and the rest of markers as independent kernels signifies the advantage of GS over traditional marker-assisted selection in utilizing whole-genome markers to account for phenotypic variance unexplainable by markers linked to QTL.

Taken together, the moderate to high predictive ability achieved for DMC and TCC in this study underpin the transformative ability of GS when adapted and integrated as a cassava breeding tool. We remark that GS offers a promise for rapid improvement of cassava for provitamin A carotenoids and DMC, especially when prediction models are properly chosen and tuned. In parallel, concerted efforts are required for further enrichment of provitamin A cassava breeding population with CBSD resistance alleles to make genomic prediction an effective tool for increasing gains for the trait. Results presented in this study complement ongoing efforts aimed at integrating the use of GS in cassava breeding programs.

## References

[jkab160-B1] Ahmar S , Gill RA, Jung KH, Faheem A, Qasim MU, et al 2020. Conventional and molecular techniques from simple breeding to speed breeding in crop plants: recent advances and future outlook. Int J Mol Sci. 21:2590. 10.3390/ijms21072590 PMC7177917 32276445

[jkab160-B2] Alicai T , Ndunguru J, Sseruwagi P, Tairo F, Okao-Okuja G, et al 2016. Cassava brown streak virus has a rapidly evolving genome: implications for virus speciation, variability, diagnosis and host resistance. Sci Rep. 6:36164. [10.1038/srep36164] 27808114 10.1038/srep36164 PMC5093738

[jkab160-B3] Alicai T , Szyniszewska AM, Omongo CA, Abidrabo P, Okao-Okuja G, et al 2019. Expansion of the cassava brown streak pandemic in Uganda revealed by annual field survey data for 2004 to 2017. Sci Data. 6:327. [10.1038/s41597-019-0334-9] 31852893 10.1038/s41597-019-0334-9 PMC6920376

[jkab160-B4] Ayinde OE , Adewumi MO, Ajewole OO, Ologunde OO. 2017. Determinants of adoption of vitamin A bio-fortified cassava variety among farmers in Oyo State, Nigeria. Croat J Food Sci Technol. 9:74–79.

[jkab160-B5] Bates D , Mächler M, Bolker B, Walker S. 2015. Fitting linear mixed-effects models using lme4. J Stat Softw. 67:1–48. 10.18637/jss.v067.i01

[jkab160-B6] Belalcazar J , Dufour D, Andersson MS, Pizarro M, Luna J, et al 2016. High-throughput phenotyping and improvements in breeding cassava for increased carotenoids in the roots. Crop Sci. 56:2916–2925.

[jkab160-B7] Bernardo R , Yu J. 2007. Prospects for genomewide selection for quantitative traits in maize. Crop Sci. 47:1082–1090.

[jkab160-B8] Berro I , Lado B, Nalin RS, Quincke M, Gutiérrez L. 2019. Training population optimization for genomic selection. Plant Gen. 12. [10.3835/plantgenome2019.04.0028] 10.3835/plantgenome2019.04.0028 PMC12810141 33016595

[jkab160-B9] Bhat JA , Ali S, Salgotra RK, Mir ZA, Dutta S, et al 2016. Genomic selection in the Era of next generation sequencing for complex traits in plant breeding. Front Genet. 7:221. 28083016 10.3389/fgene.2016.00221 PMC5186759

[jkab160-B10] Bongaarts J. 2009. Human population growth and the demographic transition. Philos Trans R Soc Lond B Biol Sci. 364:2985–2990. 19770150 10.1098/rstb.2009.0137 PMC2781829

[jkab160-B11] Bouis HE , Hotz C, McClafferty B, Meenakshi JV, Pfeiffer WH. 2011. Biofortification: a new tool to reduce micronutrient malnutrition. Food Nutr Bull. 32:S31–S40. 21717916 10.1177/15648265110321S105

[jkab160-B12] Browning BL , Zhou Y, Browning SR. 2018. A one-penny imputed genome from next-generation reference panels. Am J Hum Genet. 103:338–348. 30100085 10.1016/j.ajhg.2018.07.015 PMC6128308

[jkab160-B13] Ceballos H , Morante N, Sánchez T, Ortiz D, Aragón I, et al 2013. Rapid cycling recurrent selection for increased carotenoids content in cassava roots. Crop Sci. 53:2342–2351.

[jkab160-B14] Chávez AL , Sánchez T, Jaramillo G, Bedoya JM, Echeverry J, et al 2005. Variation of quality traits in cassava roots evaluated in landraces and improved clones. Euphytica. 143:125–133.

[jkab160-B15] Crossa J , Perez-Rodriguez P, Cuevas J, Montesinos-Lopez O, Jarquin D, et al 2017. Genomic selection in plant breeding: methods, models, and perspectives. Trends Plant Sci. 22:961–975. 28965742 10.1016/j.tplants.2017.08.011

[jkab160-B16] de los Campos G , Toro MA. 2017. Can we make genomic selection 100% accurate? J Anim Breed Genet. 134:437–437. 29164760 10.1111/jbg.12306

[jkab160-B17] de Roos AP , Hayes BJ, Goddard ME. 2009. Reliability of genomic predictions across multiple populations. Genetics. 183:1545–1553. 19822733 10.1534/genetics.109.104935 PMC2787438

[jkab160-B18] Desta Z , Ortiz R. 2014. Genomic selection: genome-wide prediction in plant improvement. Trends Pant Sci. 19:592–601. 10.1016/j.tplants.2014.05.006 10.1016/j.tplants.2014.05.006 24970707

[jkab160-B19] Endelman JB. 2011. Ridge regression and other kernels for genomic selection with R package rrBLUP. Plant Genome. 4:250–255.

[jkab160-B22] Esuma W , Kawuki RS, Herselman L, Labuschagne MT. 2016a. Stability and genotype by environment interaction of provitamin A carotenoid and dry matter content in cassava in Uganda. Breed Sci. 66:434–443. 27436954 10.1270/jsbbs.16004 PMC4902464

[jkab160-B21] Esuma W , Kawuki RS, Herselman L, Labuschagne MT. 2016b. Diallel analysis of provitamin A carotenoid and dry matter content in cassava (*Manihot esculenta* Crantz). Breed Sci. 66:627–635. 27795688 10.1270/jsbbs.15159 PMC5010302

[jkab160-B23] Eyinla TE , Maziya-Dixon B, Alamu OE, Sanusi RA. 2019. Retention of pro-vitamin A content in products from new biofortified cassava varieties. Foods. 8:177. 10.3390/foods8050177 PMC6560409 31137653

[jkab160-B1331130] Garrick DJ , Taylor JF, Fernando RL. 2009. Deregressing estimated breeding values and weighting information for genomic regression analyses. Genet. Sel. Evol. 41:55. 10.1186/1297-9686-41-55 PMC2817680 20043827

[jkab160-B24] Gegios A , Amthor R, Maziya-Dixon B, Egesi C, Mallowa S, et al 2010. Children consuming cassava as a staple food are at risk for inadequate zinc, iron, and vitamin A intake. Plant Foods Hum Nutr. 65:64–70. 20165984 10.1007/s11130-010-0157-5 PMC2840668

[jkab160-B25] Henderson K , Loreau M. 2019. An ecological theory of changing human population dynamics. People Nat. 1:31–43.

[jkab160-B26] Hickey LT , Hafeez AN, Robinson H, Jackson SA, Leal-Bertioli SCM, et al 2019. Breeding crops to feed 10 billion. Nat Biotechnol. 37:744–754. 31209375 10.1038/s41587-019-0152-9

[jkab160-B27] Hillocks RJ , Thresh JMT. 2000. Cassava mosaic and cassava brown streak virus diseases in. Africa Root. 7:1–8.

[jkab160-B28] Ikeogu UN , Davrieux F, Dufour D, Ceballos H, Egesi CN, et al 2017. Rapid analyses of dry matter content and carotenoids in fresh cassava roots using a portable visible and near infrared spectrometer (Vis/NIRS). PLoS One. 12:e0188918. 29228026 10.1371/journal.pone.0188918 PMC5724885

[jkab160-B29] Isidro-Sánchez J , Akdemir D, Montilla-Bascón G. 2017. Genome-wide association analysis using R. Methods Mol Biol. 1536:189–207. 10.1007/978-1-4939-6682-0_14 28132152 10.1007/978-1-4939-6682-0_14

[jkab160-B30] Jannink JL , Lorenz AJ, Iwata H. 2010. Genomic selection in plant breeding: from theory to practice. Brief Funct Genom. 9:166–177. 10.1093/bfgp/elq001 20156985

[jkab160-B31] Jenkins M , Byker Shanks C, Houghtaling B. 2015. Orange-fleshed sweet potato: successes and remaining challenges of the introduction of a nutritionally superior staple crop in mozambique. Food Nutr Bull. 36:327–353. 26314732 10.1177/0379572115597397

[jkab160-B32] Kandianis CB , Stevens R, Liu W, Palacios N, Montgomery K, et al 2013. Genetic architecture controlling variation in grain carotenoid composition and concentrations in two maize populations. Theor Appl Genet. 126:2879–2895. 24042570 10.1007/s00122-013-2179-5 PMC3825500

[jkab160-B33] Kawuki RS , Kaweesi T, Esuma W, Pariyo A, Kayondo IS, et al 2016. Eleven years of breeding efforts to combat cassava brown streak disease. Breed Sci. 66:560–571. 27795681 10.1270/jsbbs.16005 PMC5010303

[jkab160-B34] Kayondo SI , Pino Del Carpio D, Lozano R, Ozimati A, Wolfe M, et al 2018. Genome-wide association mapping and genomic prediction for CBSD resistance in *Manihot esculenta*. Sci Rep. 8:1549. 10.1038/s41598-018-19696-1 29367617 10.1038/s41598-018-19696-1 PMC5784162

[jkab160-B35] Kilian A , Wenzl P, Huttner E, Carling J, Xia L, et al 2012. Diversity arrays technology: a generic genome profiling technology on open platforms. Methods Mol Biol. 888:67–89. 10.1007/978-1-61779-870-2_5 22665276 10.1007/978-1-61779-870-2_5

[jkab160-B36] Kolawole PO , Agbetoye L, Ogunlowo SA. 2010. Sustaining world food security with improved cassava processing technology: the Nigeria experience. Sustainability. 2:3681–3694.

[jkab160-B37] Kristensen PS , Jahoor A, Andersen JR, Cericola F, Orabi J, et al 2018. Genome-wide association studies and comparison of models and cross-validation strategies for genomic prediction of quality traits in advanced winter wheat breeding lines. Front Plant Sci. 9:69. 10.3389/fpls.2018.00069 29456546 10.3389/fpls.2018.00069 PMC5801407

[jkab160-B38] Kulembeka HP , Ferguson M, Herselman L, Kanju E, Mkamilo G, et al 2012. Diallel analysis of field resistance to brown streak disease in cassava (*Manihot esculenta* Crantz) landraces from Tanzania. Euphytica. 187:277–288.

[jkab160-B39] Lee SH , Clark S, van der Werf JHJ. 2017. Estimation of genomic prediction accuracy from reference populations with varying degrees of relationship. PLoS One. 12:e0189775. 29267328 10.1371/journal.pone.0189775 PMC5739427

[jkab160-B40] Li D , Xu Z, Gu R, Wang P, Lyle D, et al 2019. Enhancing genomic selection by fitting large-effect SNPs as fixed effects and a genotype-by-environment effect using a maize BC1F3:4 population. PLoS One. 14:e0223898. 31622400 10.1371/journal.pone.0223898 PMC6797203

[jkab160-B41] Masumba EA , Kapinga F, Mkamilo G, Salum K, Kulembeka H, et al 2017. QTL associated with resistance to cassava brown streak and cassava mosaic diseases in a bi-parental cross of two Tanzanian farmer varieties. Theor Appl Genet. 130:2069–2090. 28707249 10.1007/s00122-017-2943-z PMC5606945

[jkab160-B42] Montagnac JA , Davis CR, Tanumihardjo SA. 2009. Nutritional value of cassava for use as a staple food and recent advances for improvement. Compr Rev Food Sci Food Saf. 8:181–194. 33467798 10.1111/j.1541-4337.2009.00077.x

[jkab160-B43] Morota G , Gianola D. 2014. Kernel-based whole-genome prediction of complex traits: a review. Front Genet. 5:363. 25360145 10.3389/fgene.2014.00363 PMC4199321

[jkab160-B44] Nassar NMA , Schwartz CA, Junior OP. 2007. Cassava diversity in Brazil: the case of carotenoid-rich landraces. Genet Mol Res. 6:116–121. 17469060

[jkab160-B45] Nielsen NH , Jahoor A, Jensen JD, Orabi J, Cericola F, et al 2016. Genomic prediction of seed quality traits using advanced barley breeding lines. PLoS One. 11:e0164494. 27783639 10.1371/journal.pone.0164494 PMC5082657

[jkab160-B46] Njoku DN , Gracen VE, Offei SK, Asante IK, Egesi CN, et al 2015. Parent-offspring regression analysis for total carotenoids and some agronomic traits in cassava. Euphytica. 206:657–666.

[jkab160-B47] Ornella L , Singh S, Perez P, Burgueño J, Singh R, et al 2012. Genomic prediction of genetic values for resistance to wheat rusts. Plant Genome. 5:136–148.

[jkab160-B48] Ozimati A , Kawuki R, Esuma W, Kayondo IS, Wolfe M, et al 2018. Training population optimization for prediction of cassava brown streak disease resistance in West African Clones. G3 (Bethesda). 8:3903–3913. 30373913 10.1534/g3.118.200710 PMC6288821

[jkab160-B49] Ozimati A , Kawuki R, Esuma W, Kayondo SI, Pariyo A, et al 2019. Genetic variation and trait correlations in an East African cassava breeding population for genomic selection. Crop Sci. 59:460–473. 33343017 10.2135/cropsci2018.01.0060 PMC7680944

[jkab160-B50] Pariyo A , Baguma Y, Alicai T, Kawuki R, Kanju E, et al 2015. Stability of resistance to cassava brown streak disease in major agro-ecologies of Uganda. J Plant Breed Crop Sci. 7:67–78. 10.5897/jpbcs2013.0490

[jkab160-B51] Perez P , de los Campos G. 2014. Genome-wide regression and prediction with the BGLR statistical package. Genetics. 198:483–495. 25009151 10.1534/genetics.114.164442 PMC4196607

[jkab160-B52] Pfeiffer WH , McClafferty B. 2007. HarvestPlus: breeding crops for better nutrition. Crop Sci. 47:S-88–S-105.

[jkab160-B53] Ray DK , Mueller ND, West PC, Foley JA. 2013. Yield trends are insufficient to double global crop production by 2050. PLoS One. 8:e66428. 23840465 10.1371/journal.pone.0066428 PMC3686737

[jkab160-B54] Sánchez T , Chávez AL, Ceballos H, Rodriguez-Amaya DB, Nestel P, et al 2006. Reduction or delay of post-harvest physiological deterioration in cassava roots with higher carotenoid content. J Sci Food Agric. 86:634–639.

[jkab160-B55] Stephenson K , Amthor R, Mallowa S, Nungo R, Maziya-Dixon B, et al 2010. Consuming cassava as a staple food places children 2-5 years old at risk for inadequate protein intake, an observational study in Kenya and Nigeria. Nutr J. 9:9. 10.1186/1475-2891-9-9 20187960 10.1186/1475-2891-9-9 PMC2837613

[jkab160-B56] Talsma EF , Melse-Boonstra A, de Kok BP, Mbera GN, Mwangi AM, et al 2013. Biofortified cassava with pro-vitamin A is sensory and culturally acceptable for consumption by primary school children in Kenya. PLoS One. 8:e73433. 24023681 10.1371/journal.pone.0073433 PMC3758265

[jkab160-B57] Turner SD. 2014. Qqman: An R Package for Visualizing GWAS Results Using Q-Q and Manhattan Plots. 10.1101/005165.

[jkab160-B58] Welsch R , Arango J, Bar C, Salazar B, Al-Babili S, et al 2010. Provitamin A accumulation in cassava (*Manihot esculenta*) roots driven by a single nucleotide polymorphism in a phytoene synthase gene. Plant Cell. 22:3348–3356. 20889914 10.1105/tpc.110.077560 PMC2990137

[jkab160-B59] Wolfe MD , Del Carpio DP, Alabi O, Ezenwaka LC, Ikeogu UN, et al 2017. Prospects for genomic selection in cassava breeding. Plant Genome. 10. doi:10.3835/plantgenome2017.03.0015 10.3835/plantgenome2017.03.0015 PMC7822052 29293806

[jkab160-B60] Xu Y , Liu X, Fu J, Wang H, Wang J, et al 2020. Enhancing genetic gain through genomic selection: from livestock to plants. Plant Commun. 1:100005. 33404534 10.1016/j.xplc.2019.100005 PMC7747995

[jkab160-B61] Xu Y , Wang X, Ding X, Zheng X, Yang Z, et al 2018. Genomic selection of agronomic traits in hybrid rice using an NCII population. Rice (NY). 11:32. 10.1186/s12284-018-0223-4 PMC5945574 29748895

[jkab160-B62] Zhang H , Yin L, Wang M, Yuan X, Liu X. 2019. Factors affecting the accuracy of genomic selection for agricultural economic traits in maize, cattle, and pig populations. Front Genet. 10:189. 30923535 10.3389/fgene.2019.00189 PMC6426750

[jkab160-B63] Zhong S , Dekkers JC, Fernando RL, Jannink JL. 2009. Factors affecting accuracy from genomic selection in populations derived from multiple inbred lines: a Barley case study. Genetics. 182:355–364. 19299342 10.1534/genetics.108.098277 PMC2674832

